# *N*-hypermannose glycosylation disruption enhances recombinant protein production by regulating secretory pathway and cell wall integrity in *Saccharomyces cerevisiae*

**DOI:** 10.1038/srep25654

**Published:** 2016-05-09

**Authors:** Hongting Tang, Shenghuan Wang, Jiajing Wang, Meihui Song, Mengyang Xu, Mengying Zhang, Yu Shen, Jin Hou, Xiaoming Bao

**Affiliations:** 1 State Key Laboratory of Microbial Technology, Shandong University, Jinan, 250100, China

## Abstract

*Saccharomyces cerevisiae* is a robust host for heterologous protein expression. The efficient expression of cellulases in *S. cerevisiae* is important for the consolidated bioprocess that directly converts lignocellulose into valuable products. However, heterologous proteins are often *N*-hyperglycosylated in *S. cerevisiae*, which may affect protein activity. In this study, the expression of three heterologous proteins, β-glucosidase, endoglucanase and cellobiohydrolase, was found to be *N*-hyperglycosylated in *S. cerevisiae*. To block the formation of hypermannose glycan, these proteins were expressed in strains with deletions in key Golgi mannosyltransferases (Och1p, Mnn9p and Mnn1p), respectively. Their extracellular activities improved markedly in the *OCH1* and *MNN9* deletion strains. Interestingly, truncation of the *N*-hypermannose glycan did not increase the specific activity of these proteins, but improved the secretion yield. Further analysis showed *OCH1* and *MNN9* deletion up-regulated genes in the secretory pathway, such as protein folding and vesicular trafficking, but did not induce the unfolded protein response. The cell wall integrity was also affected by *OCH1* and *MNN9* deletion, which contributed to the release of secretory protein extracellularly. This study demonstrated that mannosyltransferases disruption improved protein secretion through up-regulating secretory pathway and affecting cell wall integrity and provided new insights into glycosylation engineering for protein secretion.

The efficient production of recombinant therapeutic and industrial proteins is important for drug discovery and industrial applications^1^,^2^. The proteins that do not need post-translational modification can be expressed actively in a prokaryotic expression system, such as *Escherichia coli*^3^. However, the majority of heterologous proteins require post-translational modification to ensure appropriate folding and bioactivity and cannot be produced properly in prokaryotic organisms. As a eukaryotic organism, the yeast *S. cerevisiae* is able to perform post-translational modifications, such as disulfide bond formation, removal of signal peptide and *N*- and *O*-glycosylation. Thus, this yeast has been studied widely as a robust host for the expression of recombinant proteins^4^. However, the expression of heterologous proteins in *S. cerevisiae* often results in *N*-hypermannose glycosylation modification.

The glycosylation pathway modifications have been studied extensively in *Pichia pastoris*^5^. In *S. cerevisiae*, the expression of heterologous proteins often results in a hypermannose glycan structure that may result in less activity and changes to their immunogenicity^6^,^7^,^8^,^9^. Therefore, reducing hypermannose glycans may be one of the key steps for the recombinant protein production. Disruption of the genes involved in the glycosylation modification, such as Mnn2p and Mnn11p, improved the production of recombinant cellulases^10^,^11^. Deletion of the α-1,6-mannosyltransferase Och1p increased the production of the active form of human tissue-type plasminogen activator^12^. Deletion of the enzyme complex M-Pol II component Mnn10p improved secretion of multiple recombinant proteins and endogenous invertase^13^,^14^. These previous studies showed that *N*-glycosylation modification can increase protein secretion.

The *S. cerevisiae* cell wall composed of glucan polymers, chitin (β-1,4-N-acetylglucosamine polymers) and mannoproteins^15^. Mannoproteins are important for the cell wall integrity and control the cell wall porosity which allowing substances in and out the cell according to size^16^,^17^. Disruption of the mannoprotein network by deletion of cell wall mannoproteins or alteration of protein glycosylation modification can damage the cell wall integrity and increase the cell wall porosity, which often associated with the hyper-production of secretion proteins^14^,^18^,^19^,^20^. Deletion of *KRE6*, the gene encoding a β-glucan synthase required for β-1,6-glucan biosynthesis, caused the cell wall defect and enhanced the production of extracellular exo-1,3-β-glucanase which located in the periplasmic space^21^. The inactivation of Gas1p, a β-1,3-glucanosyltransferase required for cell wall assembly, led to the cell wall defect and increased the secretion of native human insulin-like growth factor 1 significantly^22^,^23^. The increase of protein secretion associated with the cell wall defect was also observed in *Trichoderma reesei*, *Hansenula polymorpha* and *Neurospora crassa*^24^,^25^,^26^.

In this study, the impact of *N*-glycosylation modification on the secretion of heterologous protein was investigated. It was found that three recombinant cellulases, Cel3A, CelA and Cel7A, were *N*-hyperglycosylated when expressed in *S. cerevisiae*. Thus, these three heterologous proteins were expressed in strains with deletions in key Golgi mannosyltransferase gene (*OCH1, MNN1* and *MNN9*) to block the formation of hypermannose glycan, and the extracellular activities of the three recombinant proteins were increased significantly. The results revealed that the improvement of extracellular activity was mainly caused by the increase in protein yield, rather than by the specific activity of the proteins. It was also observed that the transcription of key components in the secretory pathway was up-regulated and the cell wall integrity was damaged, which may be responsible for the improvement of protein production.

## Results

### Recombinant protein secretion was improved by the deletion of mannosyltransferases

**Figure 1:**
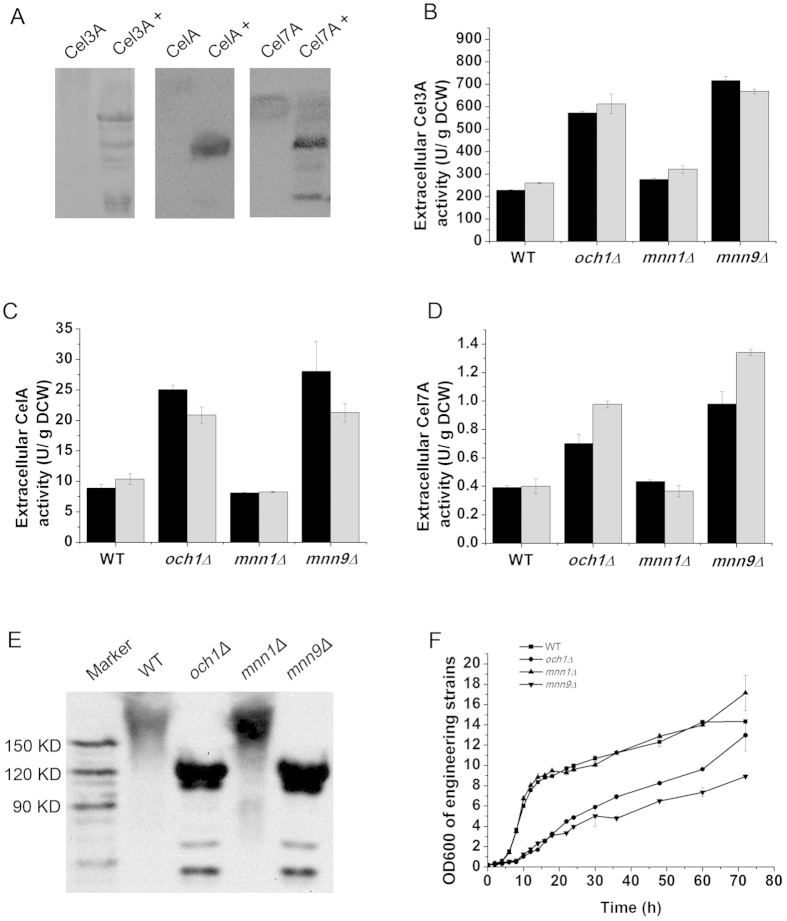
Property of recombinant proteins and growth curve of the recombinant strains. (**A**) The molecular weights of Cel3A, CelA and Cel7A with (+) or without the PNGase F treatment. For deglycosylation treatment, 48 μL denatured protein with or without 2 μL PNGase F enzyme solution (500 U/mL) was incubated in 50 mM sodium phosphate (pH 7.5) at 37 °C for 2 h. (**B**) The extracellular activity of Cel3A in recombinant strains. (**C**) The extracellular activity of CelA in recombinant strains. (**D**) The extracellular activity of Cel7A in recombinant strains. (**E**) The molecular weight of secreted Cel3A. (**F**) The growth curve of wild-type and *N*-glycosyaltion defective mutants in YPD medium. WT: wild-type strain expressing recombinant protein, *och1*Δ: *OCH1* deletion strain expressing recombinant protein, *mnn1*Δ: *MNN1* deletion strain expressing recombinant protein, *mnn9*Δ: *MNN9* deletion strain expressing recombinant protein. The data are presented as the means ± standard errors from three independent experiments.

We expressed three heterologous proteins, including *Saccharomycopsis fibuligera* β-glucosidase (Cel3A), *Clostridium thermocellum* endoglucanase (CelA) and *T. reesei* cellobiohydrolase I (Cel7A), in *S. cerevisiae*. Cel3A contains 16 potential *N*-glycosylation sites (predicted by NetNGlyc) with a theoretical molecular weight of 96.2 KD. Cel7A contains four potential *N*-glycosylation sites with a theoretical molecular weight of 57 KD. CelA contains four potential *N*-glycosylation sites with a theoretical molecular weight of 52 KD. When the secreted Cel3A, Cel7A and CelA were treated with peptide-N-glycosidase F (PNGase F), their molecular weights decreased significantly, demonstrating that the secreted Cel3A, Cel7A and CelA were *N*-hyperglycosylated (Fig. 1A). Cel3A, Cel7A and CelA were then over-expressed in the *och1*Δ, *mnn9*Δ and *mnn1*Δ strains. *OCH1* initiates the α-1,6-hypermannose glycan elongation by the first α-1,6-mannose addition, *MNN9* determines whether the Man_9_GlcNAc_2_ oligosaccharide is elongated with α-1,6-mannose backbone or stopped by the addition of α-1, 2-mannose and *MNN1* is responsible for the terminal α-1,3-mannose addition.

The extracellular activities (U/g dry cell weight (U/g DCW)) of Cel3A, CelA and Cel7A in the recombinant strains were shown in Fig. 1B–D. Deletion of *N*-glycosylation mannosyltransferases in Golgi clearly improved the extracellular activities of the recombinant proteins. Compared with the wild-type strain, the *MNN9* deletion strain increased the activity of Cel3A, CelA and Cel7A by 156%, 105% and 230%, respectively, at 72 h. Similarly, the *OCH1* deletion strain also yielded higher extracellular activities, with final activities improved by 135%, 102% and 144%, respectively. However, the disruption of *MNN1* only enhanced the Cel3A production with a 25% higher activity at 72 h. There was a similar variation trend in enzyme activity at 48 h.

Disruption of the *N*-glycosylation pathway in Golgi can block the addition of mannose to the protein hypermannose glycan and may affect the molecular weight of the protein. We therefore determined the molecular weight of the extracellular recombinant proteins. Extracellular Cel3A, CelA and Cel7A were collected from the cell culture at 72 h, and the molecular weights were determined by western blot. The molecular weights of Cel3A, CelA and Cel7A in the *OCH1* and *MNN9* deletion strains were smaller than in the wild-type strain, while the three proteins in the *MNN1* deletion strain and the wild-type strain were similar (Fig. 1E and Fig. S1A, S1B). Thus, the *N*-glycosylation modification such as the *OCH1* and *MNN9* deletions in *S. cerevisiae* indeed decreased the hypermannose glycan of recombinant proteins.

We found that the deletion of *MNN1* did not affect the growth and that the maximum specific growth rate was similar to the wild-type strain at 0.33 h^−1^; however, the deletion of *OCH1* and *MNN9* decreased the growth significantly, with the maximum specific growth rates of 0.17 h^−1^ (Fig. 1F and Table 1).

### A decrease in hypermannose glycan did not improve the activity of the recombinant proteins

**Figure 2:**
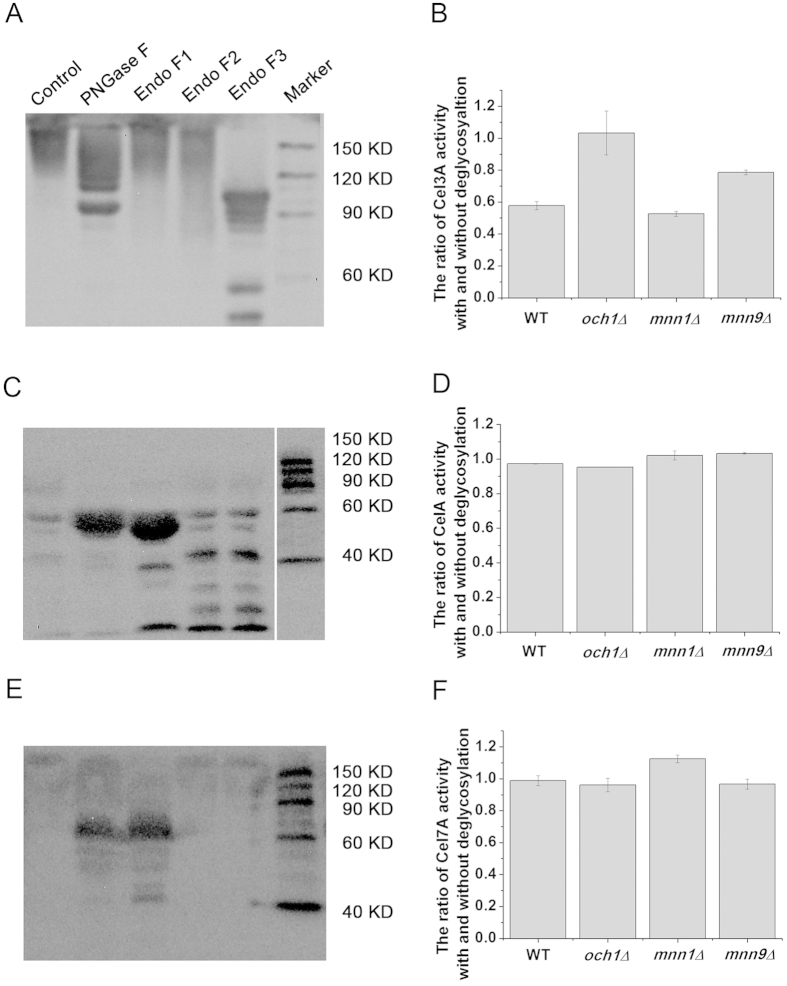
The effect of deglycosylation enzymes to the molecular weight and activity of the recombinant proteins. (**A**) The molecular weight of extracellular Cel3A with or without deglycoslylation enzyme treatment. (**B**) The ratio of extracellular Cel3A activity with or without Endo F3 treatment. Compared with the control, Endo F3 removed the *N*-glycans efficiently, thus the activity of Cel3A with and without deglycosylation by Endo F3 was measured. (**C**) The molecular weight of extracellular CelA with or without deglycosylation enzyme treatment. (**D**) The ratio of extracellular CelA activity with or without Endo F1 treatment. Compared with the control, Endo F1 removed the *N*-glycans efficiently, thus the activity of CelA with and without deglycosylation by Endo F1 was measured. (**E**) The molecular weight of extracellular Cel7A activity with or without deglycosylation enzyme treatment. (**F**) The ratio of extracellular Cel7A activity with or without Endo F1 treatment. Compared with the control, Endo F1 removed the *N*-glycans efficiently, thus the activity of Cel7A with and without deglycosylation by Endo F1 was measured. Control: samples without deglycosylation enzyme treatment. PNGase F: samples with PNGase F treatment; Endo F 1/2/3: samples with Endo F1, Endo F2 or Endo F3 treatment, respectively. The data are presented as the means ± standard errors from three independent experiments.

To determine if the decrease in hypermannose glycan affected the activity of recombinant protein, the *N*-glycans of recombinant proteins were removed, after which the changes in enzyme activity and molecular weight were compared with the control before *N*-glycan removal. PNGase F and the native protein deglycosylation kit containing endoglycosidases F1, F2 and F3 were used for the removal of protein *N*-glycans. The molecular weights of recombinant proteins Cel3A (Fig. 2A and Fig. S2A), CelA (Fig. 2C and Fig. S2B) and Cel7A (Fig. 2E and Fig. S2C) produced in the wild-type strain and the *OCH1*, *MNN1* and *MNN9* deletion strains were decreased to the theoretical molecular weights (approximately 100 KD, 50KD and 60KD, respectively) after the *N*-glycan removal. According to the decrease in molecular weight, it was observed that the *N*-glycans of Cel3A proteins could be removed effectively with endoglycosidase F3 treatment but could not be removed with PNGase F, endoglycosidase F1 or endoglycosidase F2 treatment. However, the *N*-glycans of the CelA and Cel7A proteins could be removed effectively by endoglycosidase F1 treatment. Therefore, we determined the activities of the Cel3A proteins with endoglycosidase F3 treatment, and the activities of the CelA and Cel7A proteins with endoglycosidase F1 treatment (Fig. 2B,D,F). As shown in Fig. 2B, the ratio of extracellular Cel3A activity with and without deglycosyaltion by endoglycosidase F3 treatment from the wild-type (with the complete hypermannose glycan), *MNN1* (with a relatively long hypermannose glycan) and *MNN9* (with one mannose glycan) deletion strains decreased by 42%, 45% and 28%, respectively. However, the ratio of Cel3A activity with and without deglycosyaltion from the *OCH1* deletion strain (without mannose glycan) showed a negligible change after *N*-glycan removal. This indicated that the truncation of hypermannose glycan did not improve but rather interfered with Cel3A protein activity. Moreover, the removal of the *N*-glycans with complete or truncated hypermannose glycan did not affect the ratio of CelA and Cel7A activity with and without deglycosyaltion, indicating that the *N*-glycans were not responsible for their protein activity (Fig. 2D,F). Regardless, the decrease in hypermannose glycan on the recombinant proteins as a result of the deletion of mannosyltransferases did not improve the specific activity of the proteins.

### Deletion of mannosyltransferases improved the recombinant protein yield

**Figure 3:**
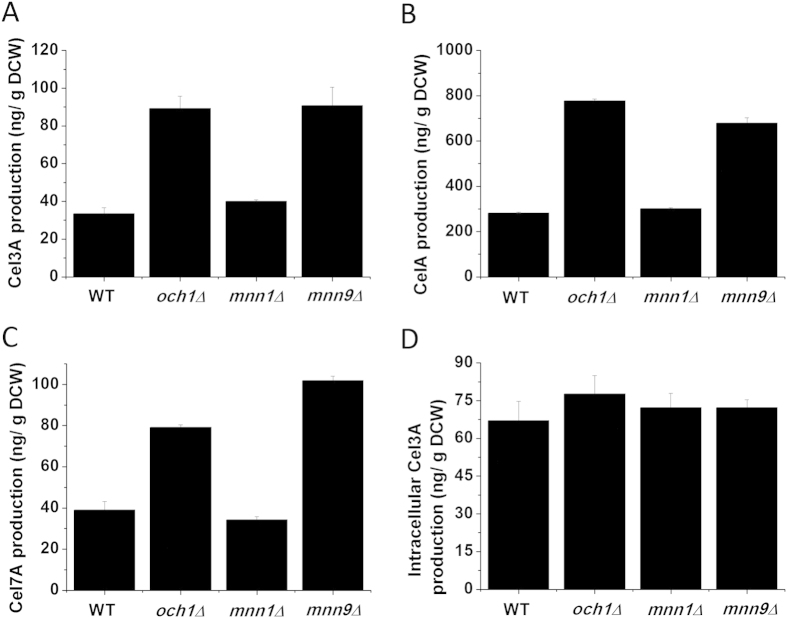
The yield of recombinant proteins. (**A**) The yield of extracellular Cel3A in recombinant strains. (**B**) The yield of extracellular CelA in recombinant strains. (**C**) The yield of extracellular Cel7A in recombinant strains. (**D**) The yield of intracellular Cel3A in recombinant strains. The data are presented as the means ± standard errors from three independent experiments.

Because the decrease in the hypermannose glycan did not improve the activity of the recombinant proteins, we determined whether the improvement in extracellular activity was attributed to the enhancement of protein yield. Compared with the wild-type strain, the *MNN9* deletion strain increased the extracellular protein yields of Cel3A, CelA and Cel7A by 170%, 140% and 160%, respectively, at 72 h. Similarly, the extracellular protein yields of Cel3A, CelA and Cel7A in the *OCH1* deletion strain was also improved by 160%, 176% and 102%, respectively (Fig. 3A–C). In addition, the *MNN1* deletion strain had a 30% higher Cel3A yield. These results revealed that the enhancement of extracellular protein yield was the main reason for the improvement in extracellular protein activity. Furthermore, the intracellular recombinant protein yield was also determined using Cel3A as an example. Compared with the wild-type strain, intracellular Cel3A production in the *OCH1*, *MNN1* and *MNN9* deletion strains increased slightly (Fig. 3D), demonstrating that the total protein production was improved by the deletion of mannosyltransferases.

### The secretory pathway was strengthened, and the UPR was not activated through the *IRE1/HAC1* pathway by the *OCH1* and *MNN9* deletions

**Figure 4:**
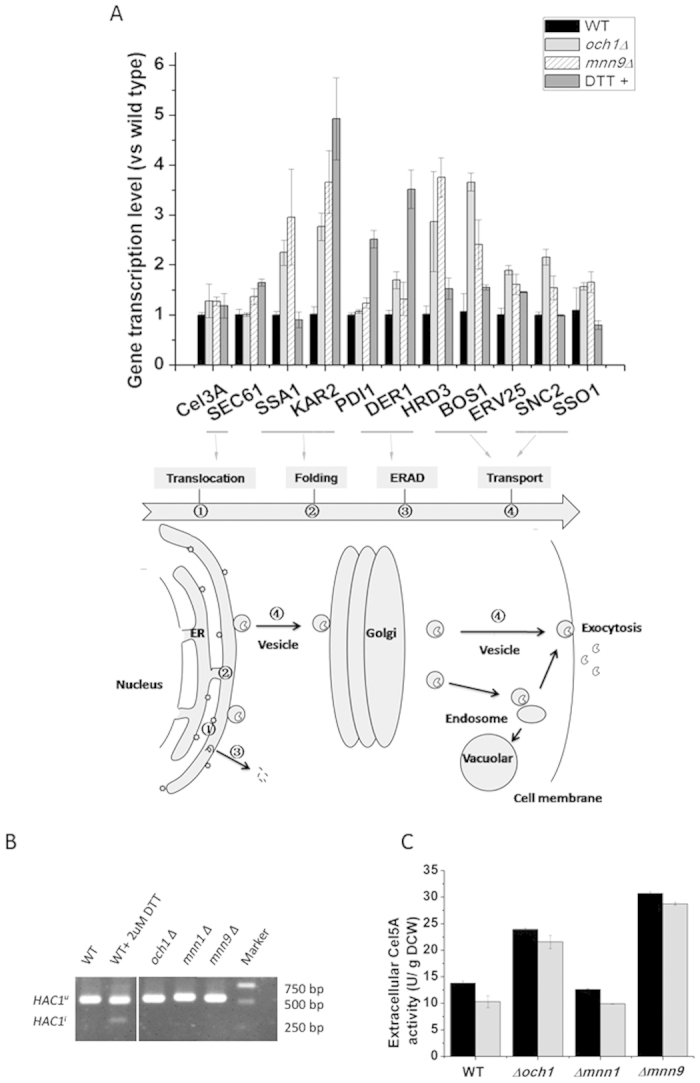
The response of the secretory pathway and the extracellular activity of Cel5A. (**A**) The relative transcription level of components involved in the secretory pathway. (**B**) *HAC1* mRNA splicing levels in wild-type, *N*-glycosyaltion defective mutants and DTT treated cells (DTT+). DTT+ was used as the positive controls. *HAC1*^*u*^ denotes unspliced *HAC1* mRNA, and *HAC1*^*i*^ denotes induced (spliced) *HAC1* mRNA. (**C**) The activity of extracellular Cel5A (a non-glycosylated protein, a *N*-glycosylation site mutation rCel5A (rN124D) of *Trichoderma reesei* endoglucanase Cel5A) in recombinant strains. The data are presented as the means ± standard errors from three independent experiments.

Because the efficiency of the protein secretory pathway largely affects recombinant protein production, we therefore determined whether the secretory pathway was affected by the *OCH1* and *MNN9* deletions. The transcription of key genes in the secretory pathway involved in protein translocation, folding, ER-associated degradation (ERAD) and vesicle trafficking was determined. The results showed that protein folding-related genes, such as *KAR2* and *SSA1*, protein trafficking-related genes, including *BOS1*, *ERV25*, *SNC2* and *SSO1*, and genes involved in ERAD, such as *DER1* and *HRD3* were up-regulated by the *OCH1* and *MNN9* deletions. However, *SEC61*, the gene encoding a protein involved in protein translocation, and *PDI1*, encoding protein disulfide isomerase involved in the formation of disulfide bonds, were unaffected (Fig. 4A). The results indicated that enhancement of the secretory pathway by *OCH1* and *MNN9* deletions may contribute to improvement in recombinant protein production.

We further investigated whether the up-regulation of the secretory pathway was caused by the activation of the UPR. The accumulation of unfolded or misfolded proteins induces ER stress and triggers the UPR. The transcription factor Hac1p, generating from spliced *HAC1* mRNA through the intron removal of unspliced *HAC1* mRNA, can regulate UPR by up-regulating the transcription of many genes in the secretory pathway^27^,^28^. The *HAC1* splicing level was used as an indicator of activation of the UPR. DTT disturbs the formation of protein disulfide bond and is known to induce the UPR. Compared with the *OCH1* and *MNN9* deletion strains, the DTT-treated wild-type strain up-regulated not only *KAR2*, *BOS1*, *ERV25*, *DER1* but also *SEC61* and *PDI1*, while *SNC2* and *SSO1*, involved in vesicle transport from Golgi to membrane, were not affected. The level of spliced *HAC1* mRNA was assessed, and accumulation of spliced *HAC1* (*HAC1*^*i*^) was observed in the DTT-treated strain, but not in the glycosylation disruption strains (Fig. 4B). This demonstrated that the up-regulation of the secretory pathway was not induced by the UPR through the classic *IRE1*/*HAC1* pathway in *OCH1* and *MNN9* deletion strains.

To investigate whether the mannosyltransferase deletion also affect the secretion of non-glycosylated protein, we expressed a non-glycosylated protein, a *N*-glycosylation site mutation rCel5A (rN124D) of *Trichoderma reesei* endoglucanase Cel5A in the mannosyltransferase deletion strains^29^. As shown in Fig. 4C, the secretion of rCel5A was also improved significantly in *OCH1* and *MNN9* deletion strains, indicating that the enhancement of protein secretion was not caused by the change of glycan pattern on the secreted protein, but may be caused by the strengthen of secretory pathway.

### *OCH1* and *MNN9* deletions caused cell wall integrity defects

**Figure 5:**
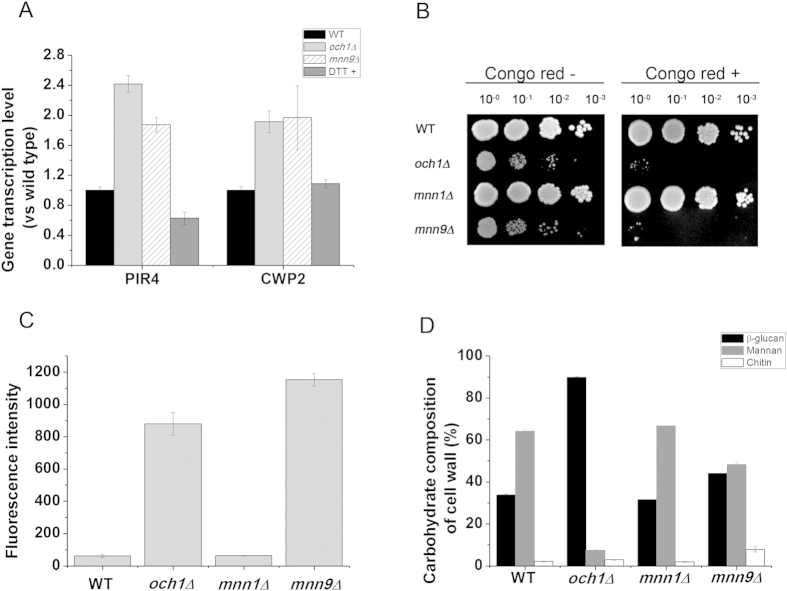
The defects of cell wall integrity. (**A**) The relative transcription levels of cell wall proteins in recombinant strains and DTT treated strain (DTT+). (**B**) Congo red sensitivity assay. The cell density was normalized to OD_600_ of 1.0 and diluted four times in a 10-fold series (10^0^, 10^−1^, 10^−2^ and 10^−3^). Congo red –: cultivation without the addition of Congo red; Congo red+: cultivation with the addition of 5 μg/ml Congo red. (**C**) The fluorescence intensity of recombinant strains after staining with CFW. (**D**) Carbohydrate composition of cell wall of wild-type and *N*-glycosyaltion defective mutants. The data are presented as the means ± standard errors from three independent experiments.

The cell wall mannoproteins are important components for the synthesis of cell wall. To study whether the cell wall integrity was affected by mannosyltransferase deletion, the transcription of the genes *PIR4* and *CWP2*, encoding cell wall mannoproteins, was determined, which showed an up-regulation in the *OCH1* and *MNN9* deletion strains, but not in the DTT-treated strain (Fig. 5A). Meanwhile, the *OCH1* and *MNN9* deletion strains were more sensitive to the cell wall-perturbing materials, such as Congo red and CFW and glucan-digesting enzyme lyticase (Fig. 5B,C and Fig. S3A). Moreover, the carbohydrate composition of cell wall of stationary-phase cells was also determined and the results showed that the percentage of mannan decreased sharply in *OCH1* and *MNN9* deletion strains (Fig. 5D). The hypermannose glycan of cell surface glycoproteins determines the cell wall porosity, which limits the penetrability of the cell wall^19^. Thus, the disruption of protein glycosylation may affect the cell wall porosity. The results showed that the relative cell wall porosity of the *OCH1* and *MNN9* deletion strains was clearly higher than that of the wild-type strain (Table 2). These results demonstrated that the cell wall integrity was disrupted in the *OCH1* and *MNN9* deletion strains.

**Figure 6:**
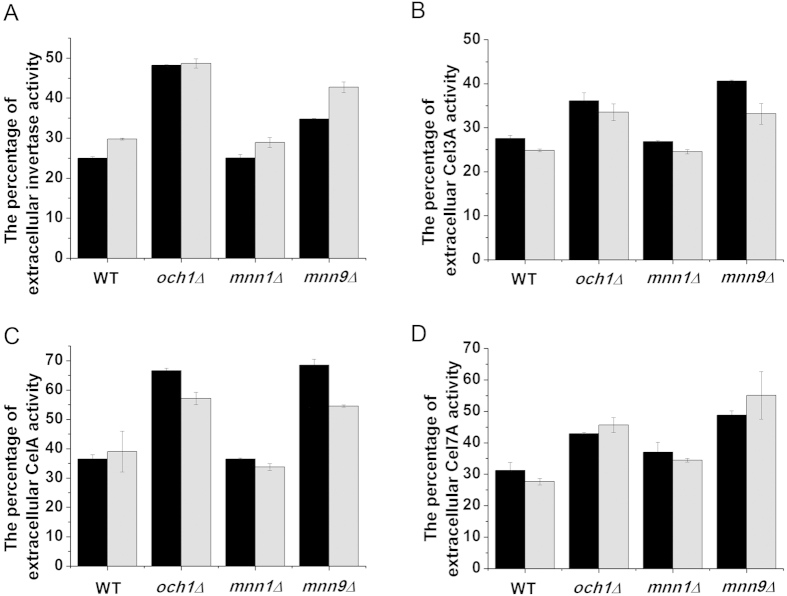
The percentage of the extracellular protein activity (extracellular protein activity/the activity of extracellular and periplasmic protein). (**A**) The percentage of the extracellular invertase activity. (**B**) The percentage of the extracellular Cel3A activity. (**C**) The percentage of the extracellular CelA activity. (**D**) The percentage of the extracellular Cel7A activity. The data are presented as the means ± standard errors from three independent experiments.

Invertase, a yeast endogenous secreted protein, mainly accumulates in the periplasm. In the *OCH1* and *MNN9* deletion strains, the percentage of extracellular invertase (extracellular activity/extracellular activity and periplasmic activity) increased obviously, while the percentage was not changed in the *MNN1* deletion strain (Fig. 6A). It was also observed that the percentage of the extracellular activity of these three recombinant proteins was also increased in the *OCH1* and *MNN9* deletion strains (Fig. 6B–D). These results demonstrated that the disruption of cell wall integrity contributed to greater protein secretion into the extracellular matrix.

**Figure 7:**
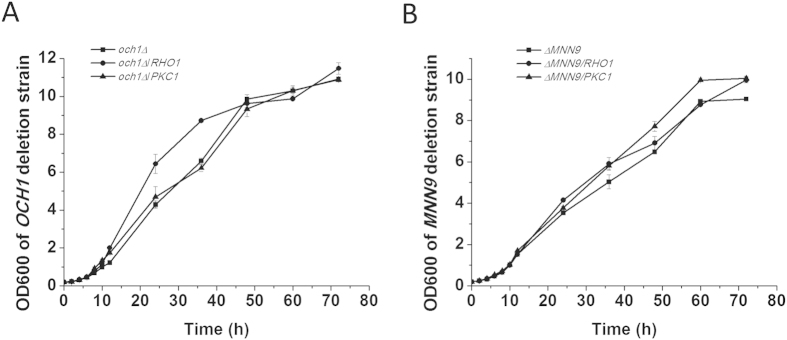
The growth curve of the *RHO1*- and *PKC1*-expressing strains. (**A**) The growth curve of the *OCH1* deletion strain expressing *RHO1* and *PKC1*. (**B**) The growth curve of the *MNN9* deletion strain expressing *RHO1* and *PKC1*. The data are presented as the means ± standard errors from three independent experiments.

A defect in the cell wall can lead to a sensitivity to osmotic stress and may be one of the reasons for the growth deficiency of the *OCH1* and *MNN9* deletion strains. However, growth was not suppressed by sorbitol addition (data not shown). In addition, a cell wall defect can also activate the cell wall integrity signaling pathway to regulate cell wall remodeling for survival. The genes *RHO1* and *PKC1*, involving in cell wall integrity signaling pathway, were over-expressed to strengthen the cell wall integrity^30^,^31^. In Rho1p and Pkc1p over-expressing strains, the growth defect of the *OCH1* and *MNN9* deletion strains was suppressed slightly (Fig. 7 and Table 1). In addition, the sensitivity of Congo red and CFW in Rho1p and Pkc1p over-expressing strains was partially decreased (Fig. S3B,C), indicating that the defect in cell wall integrity was partially suppressed.

## Discussion

In this study, the recombinant proteins Cel3A, CelA and Cel7A were found to be hyperglycosylated with significant increases in their molecular weights, even though CelA was from a prokaryotic organism. This indicated that heterologous proteins with potential glycosylation sites may be modified by the yeast glycosylation process regardless of their origin. Therefore, Cel3A, CelA and Cel7A were expressed in the Golgi mannosyltransferase deletion strains that blocked the extension of the outer chain to avoid hyperglycosylation. The extracellular activities of the three recombinant proteins were enhanced significantly. Moreover, the molecular weights of the proteins secreted from the *OCH1* and *MNN9* deletion strains were clearly reduced. These results showed that the disruption of the key mannosyltransferases in the Golgi could successfully block the *N*-hypermannose glycan extension and increase the extracellular activity of recombinant proteins. This improvement in extracellular activity could result from the increase in protein specific activity or protein yield.

Removal of the *N*-glycans without the mannose outer chain in the *OCH1* deletion strain did not affect the Cel3A activity, while removal of the *N*-glycans with a complete or truncated mannose outer chain in wild-type, *MNN1* and *MNN9* deletion strains decreased the Cel3A activity. The results demonstrated that some heterologous proteins required the outer chain of *N*-hypermannose glycan to ensure their activity and that truncation of the *N*-hypermannose glycan might not enhance the protein activity but rather have a negative effect. In contrast, the removal of *N*-glycans from CelA and Cel7A proteins had no effect on their activities, indicating that the hypermannose glycan of some recombinant proteins did not disturb or enhance their protein activity. In conclusion, the truncation of the hypermannose glycan of recombinant proteins in *S. cerevisiae* did not improve their protein specific activity.

The results demonstrated that improvement in the extracellular protein activity was mainly due to an increase in the protein production. Recombinant protein production is largely affected by the efficiency of the protein secretory pathway. Over-expression of key genes involved in protein folding, such as *KAR2* and *PDI1*, and vesicle trafficking, such as *SSO1* and *SNC1* can strengthen the secretory pathway and enhance the secretion of heterologous proteins^32^,^33^. In the mannosyltransferase deletion strains, many genes in the secretory pathway, including protein folding-related genes such as *KAR2* and *SSA1*, protein trafficking genes such as *BOS1*, *ERV25*, *SNC2* and *SSO1*, and the genes involved in ERAD such as *DER1* and *HRD3*, were up-regulated. Thus, improvement in protein yield might be caused by the enhancement of the secretory pathway.

The UPR can regulate the secretory pathway by up-regulating the transcription of many genes in the secretory pathway^34^. The genes involved in protein translocation, such as *SEC61*, protein folding, such as *KAR2* and *PDI1*, vesicle trafficking, such as *BOS1* and *ERV25*, and ERAD, such as *DER1* and *HRD3*, were up-regulated in the DTT-treated wild-type strain. However, the genes involved in vesicle trafficking from the Golgi to the membrane, such as *SSO1* and *SNC2*, were not affected. These results were consistent with a previous study, in which the genes in the secretory pathway were up-regulated by the UPR^34^. The spliced *HAC1* mRNA was induced in the DTT-treated strain but not in mannosyltransferase deletion strains. Thus, although the genes up-regulated in the secretory pathway through the *OCH1* and *MNN9* deletion partially overlapped with the UPR targets, this was induced in an *IRE1*/*HAC1*-independent manner.

In a previous study, the *OCH1* deletion resulted in a defect in cell wall integrity and triggered the mitogen-activated protein kinase (MAPK) pathway^31^. Chen *et al.* reported that MAPK pathway was activated during ER stress and regulated ER stress in an *IRE1*/*HAC1*-independent manner^35^. Scrimale *et al.* reported that a defect in cell wall integrity caused ER stress and induced the UPR by Hac1p and that this activation was regulated by signaling through the cell wall integrity MAPK pathway^36^. These reports suggested that a regulatory relationship might exist between cell wall integrity and the secretory pathway. In our study, both *OCH1* and *MNN9* deletions could up-regulate the components of the secretory pathway. In addition, the *OCH1* and *MNN9* deletions affected the cell wall integrity. We therefore speculated that the up-regulation of the secretory pathway might correlate with the cell wall defect. However, the relation bewteen cell wall defect and secretory pathway regulation is still not very clear and needs to be investigated further.

A defect in cell wall integrity may be one reason for severe growth deficiency of the *OCH1* and *MNN9* deletion strains. It is reported that the growth defect of the *OCH1* deletion strains can be partially suppressed by an osmotic stabilizer and by the over-expression of Pkc1p, which is involved in the cell wall integrity signaling pathway^31^. Pkc1p and Rho1p, which is considered to be the master regulator of the cell wall integrity signaling pathway, slightly recovered the growth defect of the *OCH1* and *MNN9* deletion strains and the defect in cell wall integrity was partially suppressed by the over-expression of Rho1p and Pkc1p^30^,^37^. However, the osmotic stabilizer sorbitol did not suppress the growth deficiency of the *OCH1* and *MNN9* deletion strains. These results indicated that the defect in cell wall integrity might not be the only damaging factor responsible for the growth defect in the *OCH1* and *MNN9* deletion strains^38^.

In this study, we found that recombinant proteins with potential glycosylation sites were hyperglycosylated and that disruption of the hypermannose glycan extension in the Golgi significantly improved recombinant protein production. This improvement was mainly due to the enhancement of protein yield rather than to an increase in protein activity. The secretory pathway was strengthened in the *OCH1* and *MNN9* deletion strains in a *HAC1*-independent manner, which may contribute to the improved production of recombinant proteins. However, the relationship between glycosylation disruption and secretory pathway regulation is still unclear; further work should be carried out to investigate how glycosylation disruption affects the secretory pathway.

## Materials and Methods

### Media and growth conditions

*Escherichia coli* Trans5α (TransGene Biotech) was used for the propagation of plasmids construction. *S. cerevisiae* CEN.PK102-3A was used as the host for the expression of heterologous protein^39^. *E. coli* Trans5α was cultivated in LB medium (5 g/L yeast extract, 10 g/L peptone, 10 g/L glucose). CEN.PK102-3A was grown in YPD medium (10 g/L yeast extract, 20 g/L peptone, 20 g/L glucose). The recombinant strains were cultivated in SC-SCAA medium as previously described^40^. The cultivation of recombinant strains was performed at 30 °C, 200 rpm in 100-ml flasks with a 40 ml working volume. The initial OD_600_ (optical density) was 0.2.

### Recombinant plasmids and strains construction

The recombinant strains and plasmids involved in this study were listed in Table S1. The primers were shown in Table S2. The ligation was performed using the Gibson assembly^41^. The *Cel3A* gene with its native signal sequence and FLAG-tagged sequence was amplified from the plasmid pTH-BGL, the *CelA* gene with an *INU1* signal sequence and myc-tagged sequence was amplified from pTH-CEL (37) and each gene was then ligated into the plasmid PYX242WS^42^ under the control of the *TPI1* promoter and the *PGK1* terminator. The gene of Cel5A from *T. reesei* with a site mutation (rN124D) was amplified from recombinant plasmid pAJ401-cel5A-N124D^29^ and inserted into plasmid PYX242WS. The yeast centromere plasmid pJFE1 containing the *URA3* gene as a marker was used to over-express the genes *RHO1* and *PKC1*. *RHO1* and *PKC1* were amplified from the genomic DNA of CEN.PK102-3A and ligated into the pJFE1 plasmid^43^ under the control of the *TEF1* promoter and the *PGK1* terminator.

The *OCH1*, *MNN9* and *MNN1* were each deleted in CEN.PK102-3A as described previously^44^, resulting in the *och1*Δ, *mnn9*Δ and *mnn1*Δ strains. The plasmids pBGL, pCelA, pCel5A and pYX242WS were used to transform CEN.PK102-3A, *och1*Δ, *mnn9*Δ and *mnn1*Δ. The plasmids pJFE1, pRHO and pPKC were then transformed into the Cel3A-expressing strains.

### Protein quantification

The cell culture was centrifuged at 18,000 g for 4 min. The supernatant was collected and the cells were washed and resuspended in 50 mM citrate buffer (pH 5.0) for the measurement of enzyme activity. For extraction of periplasmic and intracellular proteins, the cells were washed with Z buffer (60 mM Na_2_HPO_4_, 40 mM NaH_2_PO_4_, 10 mM KCl, 1 mM MgSO_4_, 50 mM β-mercaptoethanol, pH 7.0). The cells were then resuspended in 1 mL Z buffer with 0.1 mg/mL of lyticase (Sigma, USA) to an OD_600_ of 5.0 and incubated at 20 °C for 3 hours to remove the cell walls. After incubation with lyticase, the samples were centrifuged at 18,000 g for 4 min and the supernatant were collected to measure the periplasmic enzymes activity. The cellular proteins were extracted from the lysate of the cell pellets, which were broken by a Precellys 24 Homogenizer (Bertin Technologies, France) with glass beads. The protein titer of the recombinant proteins was measured by Flag-tag and myc-tag ELISA detection kits (Biovol, Shanghai, China) in accordance with the manufacturer’s instruction.

β-glucosidase activity was determined as described previously using *p*-nitrophenyl-β-D-glucopyranoside *p*NPG (Sigma, USA) as the substrate^45^. The enzyme was incubated in 50 mM citrate buffer (pH 5.0) with 5 mM *p*NPG at 50 °C for 30 min. The reaction was stopped by adding 10% sodium carbonate, and *p*-nitrophenol released from *p*NPG was determined at 405 nm. The endoglucanase activity assay was quantified using carboxymethylcellulose sodium salt as the substrate (Sigma, USA)^46^. Reducing sugars were detected at 540 nm after boiling with the dinitrosalicylate (DNS) reagent for 10 min. Cellobiohydrolase activity was determined using *p*-nitrophenyl-b-D-cellobioside (*p*NPC) (Sigma, USA) as the substrate as described previously. The enzyme was incubated in 50 mM citrate buffer (pH 5.0) with 1.67 mM *p*NPG at 50 °C for 30 min. The reaction was stopped by adding 10% sodium carbonate, and the *p*-nitrophenol released from *p*NPC was determined at 405 nm^47^. Invertase activity secreted into the periplasmic space was measured as described previously^33^. Glucose released from sucrose was quantified using a D-GLUCOSE (GOPOD) kit (Megazyme K-CERA, Wicklow, Ireland).

### Analysis of cell wall integrity in recombinant strains

Congo red sensibility was monitored by a qualitative growth assay on plates containing Congo red as described previously with some modification^48^. Briefly, the cells were grown overnight and harvested by centrifugation. The cell pullets were washed three times and resuspended in sterile H_2_O. The cell density was normalized to an OD_600_ of 1.0 and diluted four times in a 10-fold series (OD_600_ 10^−1^, 10^−2^, 10^−3^, 10^−4^). Four μl of each dilution of the samples was spotted onto the plate with or without 50 ug/mL Congo red.

The lyticase sensibility assay was performed as previously described with some modification^48^. Cells were harvested by centrifugation, washed three times and resuspended in 10 mM Tris-HCl (pH = 7.4) with an approximate OD_600_ of 1.5. The cells were then treated at 30 °C with 0.5 U lyticase (Sigma, USA). The residual cells that were not lysed were monitored by OD_600_.

For cell wall staining, Calcofluor white (CFW) (Sigma, USA) was used as described previously^49^. Cells at an approximate OD_600_ of 1.5 were harvested by centrifugation, and washed three times and resuspended in sterile water with 100 μg/mL CFW. The solution was incubated at 30 °C for 2 min, harvested, washed twice with sterile water, and resuspended in 1 ml of water before measuring the fluorescence intensity at 355 nm/460 nm.

The relative cell porosity assay was determined as described previously^50^. The cells were harvested at 36 h, and washed with 10 mM Tris-HCl (pH = 7.4). The cells were then resuspended in 10 mM Tris-HCl (pH = 7.4) containing 5 ug/mL DEAE-dextran (a large hydrodynamic radius causing only limited cell leakage due to limited passage through the cell wall) or 10 ug/mL poly-L-lysine (a small hydrodynamic radius caused cell leakage independent of cell wall porosity) (Songon Biological Engineering, China) to an density of 10 OD_600_/mL and incubated at 30 °C for 30 min. Subsequently, the samples were centrifuged at 18,000 g, after which the supernatant was filtered and the UV-absorbing compounds leaking from the cells as a result of by the polycation treatment were measured at 260 nm. The ratio between DEAE-dextran- and poly-L-lysine-induced cell leakage was calculated to determine the relative cell wall porosity.

### Isolation of cell wall and analysis of cell wall carbohydrate

The isolation of cell wall and analysis of carbohydrate composition was performed as previously described^51^. In brief, cells were harvested during stationary-phase at 48 h and washed once with deionized water. The cell pellet was resuspended with 1 ml TE buffer and broken with 1 g acid-washed glass beads. The supernatant was transferred into a 15 ml falcon tube. The glass beads were washed five times and the washing solutions were also collected in 15 ml falcon tube. Samples were centrifuged at 4,800 g for 15 min and the pellets were resuspended in 1 mL TE buffer for centrifugation at 3,000 g for 5 min. The supernatant was dried at 110 °C. Cell walls were hydrolyzed with 72% H_2_SO_4_ and the carbohydrate was analyzed by CarboPac^TM^ PA1 anion-exchange column (2 × 250 mm, Dionex). Elution was performed with 18 mM NaOH at a flow rate of 0.5 mL/min.

### Extracellular protein concentration and PNGase F treatment

The extracellular protein from the cell cultures were concentrated using the Amicon ultra 15 mL centrifugal filter with an NMWL membrane of 30 KD (Millipore, Germany) and then washed three times with sterilized water. *N*-linked oligosaccharides of the recombinant proteins were removed using PNGase F or the native protein deglycosylation kit containing endoglycosidase F1, F2 and F3 (Sigma, USA) according to the manufacturer’s instructions. The proteins were then used for the activity assay and western blot analysis.

### Western blot

SDS-PAGE and western blots were performed as described previously^37^. The recombinant proteins were detected using rabbit polyclonal octA-, his- or myc-probe antibodies (Santa Cruz Biotechnology, USA) as the primary antibody and a horseradish peroxidase-labeled goat anti-rabbit IgG as the secondary antibody.

### *HAC1* mRNA splicing and real-time quantitative PCR (qPCR)

Stains grew in 40 mL of SC-SCAA medium at 30 °C at 200 rpm from OD 0.2 to OD_600_ 0.6, and cells were harvested and washed. Total RNA was extracted using the UNIQ-10 Trizol RNA extraction kit (Sangon Biological Engineering, China). The PrimeScript RT-PCR Kit (Takara, Japan) was used for cDNA synthesis. The relative transcription levels of the genes were quantified by real-time quantitative PCR using the SYBR Green Master Mix Kit (Roche Molecular Biochemicals, Germany). *HAC1* mRNA splicing were detected through PCR using cDNA as the template.

## Additional Information

**How to cite this article**: Tang, H. *et al. N*-hypermannose glycosylation disruption enhances recombinant protein production by regulating secretory pathway and cell wall integrity in *Saccharomyces cerevisiae*. *Sci. Rep.* **6**, 25654; doi: 10.1038/srep25654 (2016).

## Supplementary Material

Supplementary Information

## Figures and Tables

**Table 1:** The maximum specific growth rate of the recombinant protein production strains.

Strain	*μ*_max_^a^ (h^−1^)			
	Wild type	Δ*OCH1*	Δ*MNN1*	Δ*MNN9*
Recombinant strains	0.32	0.17	0.31	0.17
Rho1p-expressing strains	0.30	0.19	0.30	0.17
Pkc1p-expressing strains	0.33	0.20	0.33	0.18

^a^Maximum specific growth rate.

The data are presented as the means ± standard errors from three independent experiments. The standard errors are less than 1%.

**Table 2:** The cell wall porosity as determined by the polycation assay.

	Wild type	Δ*OCH1*	Δ*MNN1*	Δ*MNN9*
Porosity	8.8 ± 0.56	49.7 ± 1.15	11.1 ± 0.35	28.1 ± 2.05
