# Supplementary material for: N-hypermannose glycosylation disruption enhances recombinant protein production by regulating secretory pathway and cell wall integrity in Saccharomyces cerevisiae
Source: Sci Rep. 2016 May 9;6:25654. doi: 10.1038/srep25654 (PMC4860636; doi:10.1038/srep25654)
Supplement: Supplementary Information [file srep25654-s1.doc]

***N*-hypermannose glycosylation disruption enhances recombinant protein production by regulating secretory pathway and cell wall integrity in** ***Saccharomyces cerevisiae***

Hongting Tang, Shenghuan Wang, Jiajing Wang, Meihui Song, Mengyang Xu, Mengying Zhang, Yu Shen, Jin Hou*, Xiaoming Bao*

***
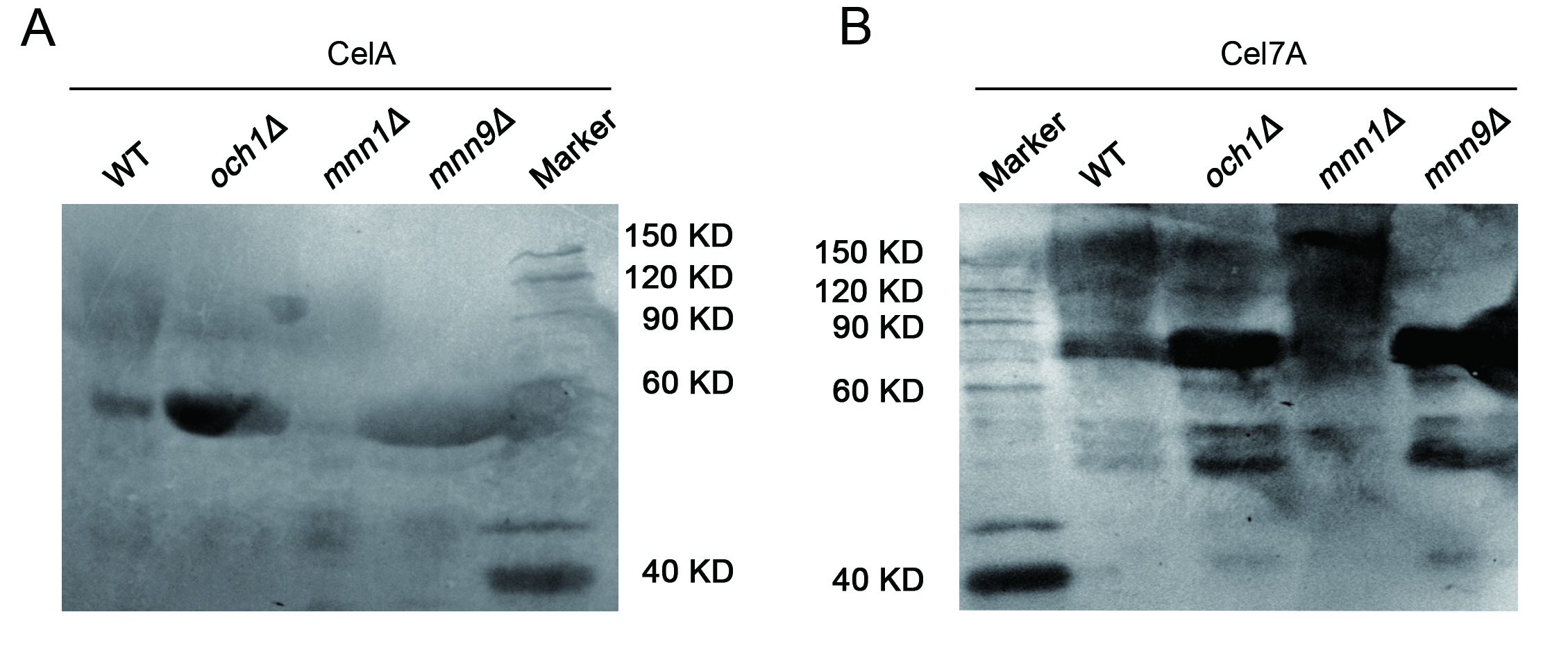
***

Fig. S1 The molecular weight of the recombinant proteins. (A) The molecular weight of secreted CelA. (B) The molecular weight of secreted Cel7A. The data are presented as the means ± standard errors from three independent experiments.


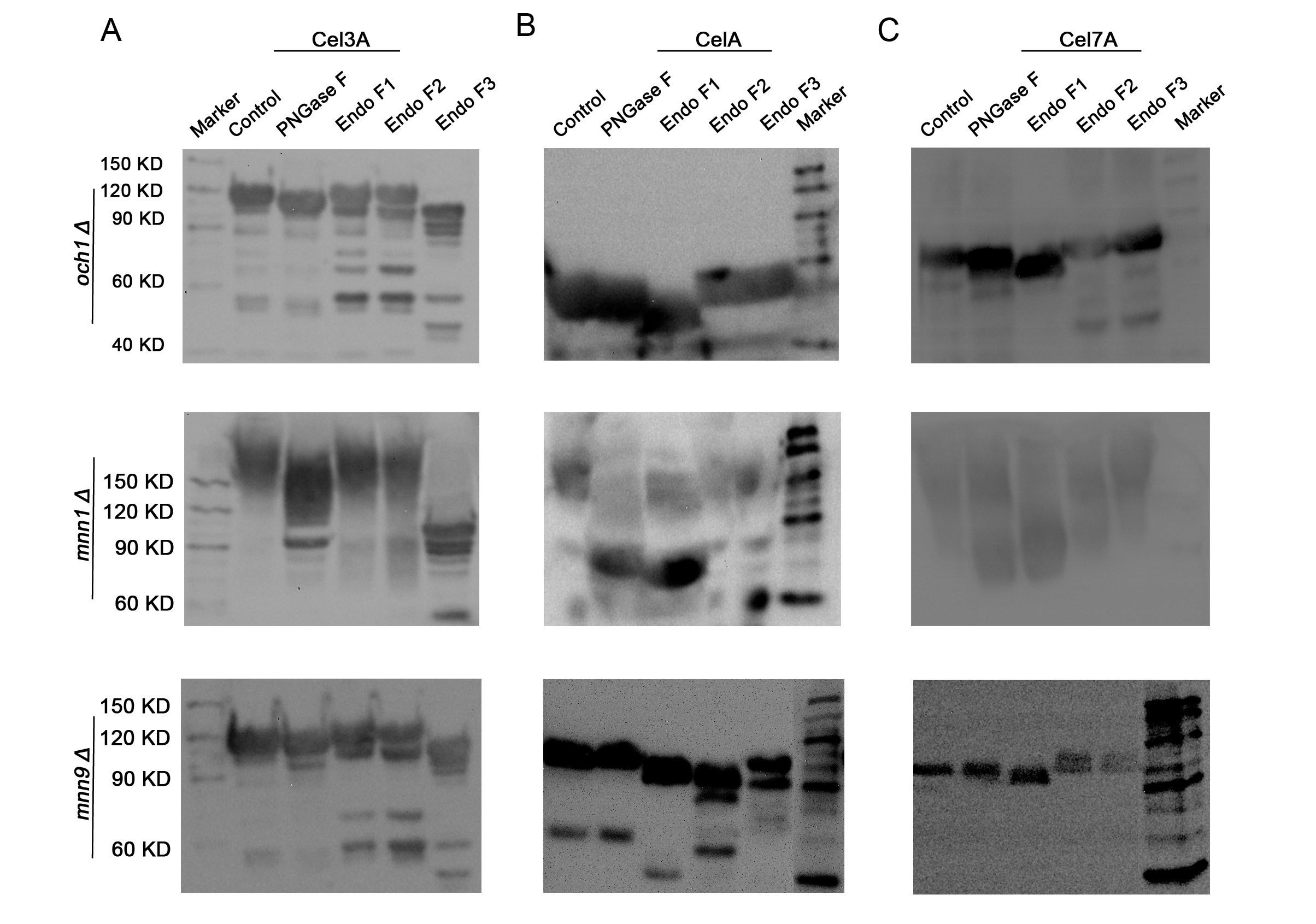


Fig. S2 The molecular weight of extracellular recombinant protein with treatment with PNGase F or Endo F1, 2, 3. (A) The molecular weight of extracellular Cel3A. (B) The molecular weight of extracellular CelA. (C) The molecular weight of extracellular Cel7A. *ochΔ*: *OCH1* deletion strains; *mnn1Δ*: *MNN1* deletion strains; *mnn9Δ*: *MNN9* deletion strains. Control: samples not treated with deglycosylation enzymes. PNGase F: samples treated by PNGase F; Endo F 1/2/3: samples treated by Endo F1, Endo F2 or Endo F3. The data are presented as the means ± standard errors from three independent experiments.


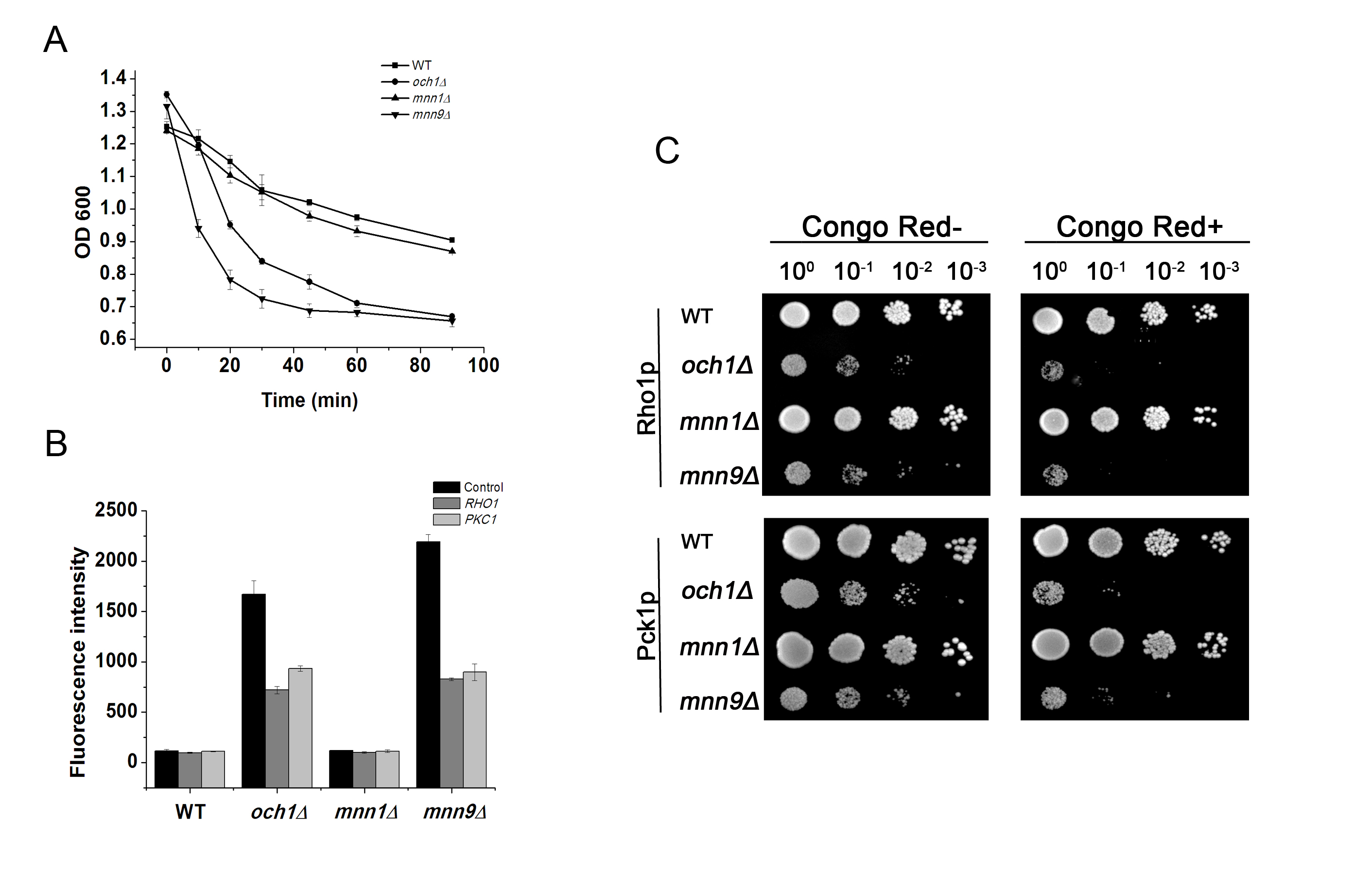


Fig. S3 The defect of cell wall integrity. (A) The glucan-digesting enzyme sensibility assay. (B) The fluorescence intensity with staining CFW of Rho1- and Pkc1p-expressing stains. (C) Congo red sensibility assay of Rho1- and Pkc1p-expressing stains. The data are presented as the means ± standard errors from three independent experiments.

Table S1. Plasmids and strains used in this study.

| Plasmid | Genotype | Reference |
| --- | --- | --- |
| PYX242WS | Yeast 2μ plasmid with *LEU2* marker | (41) |
| pJFE1 | Yeast centromere plasmid with *URA3* marker | (42) |
| pBGL | PYX242WS with *TPI1* promoter, *PGK1* terminator and *Cel3A* gene from *S. fibuligera* | This study |
| pCelA | PYX242WS with *TPI1* promoter, *PGK1* terminator and *CelA* gene from *C. thermocellum* | This study |
| pCel5A | PYX242WS with *TPI1* promoter, *PGK1* terminator and r*Cel5A* gene from *T. reesei* | This study |
| pRHO | pJFE1 with *TEF1* promoter, *PGK1* terminator and *RHO1* gene | This study |
| pPKC | pJFE1 with *TEF1* promoter, *PGK1* terminator and *PKC1* gene | This study |
| Strain |  |  |
| CEN.PK102-3A | *MATa ura3-52 leu2-112* | (37) |
| BGL /CelA/Cel5A | CEN.PK102-3A derivative; pBGL (pCelA, pCel5A) | This study |
| BOCH1 /AOCH1/5OCH1 | CEN.PK102-3A derivative; *OCH1*::*KanMX4*/pBGL (pCelA, pCel5A) | This study |
| BMNN9 /AMNN9/5MNN9 | CEN.PK102-3A derivative; *MNN9*::*KanMX4*/ pBGL (pCelA, , pCel5A) | This study |
| BMNN1 /AMNN1/5MNN1 | CEN.PK102-3A derivative; *MNN1*::*KanMX4*/ pBGL (pCelA, pCel5A) | This study |
| *cel7AF* | CEN.PK102-3A derivative; pJCF | (43) |
| *Och1Δ*/*cel7AF* | CEN.PK102-3A derivative; *OCH1:: KanMX*/ *pJCF* | (43) |
| *mnn1Δ*/*cel7AF* | CEN.PK102-3A derivative; *MNN1:: KanMX*/ *pJCF* | (43) |
| *mnn9Δ*/*cel7AF* | CEN.PK102-3A derivative; *MNN9:: KanMX*/ *pJCF* | (43) |
| rBGL/pBGL | BGL derivative; pRHO/pPKC | This study |
| rBOCH1/pBOCH1 | BOCH1 derivative; pRHO/pPKC | This study |
| rBMNN9/pBMNN9 | BMNN9 derivative; pRHO/pPKC | This study |
| rBMNN1/pBMNN1 | BMNN1 derivative; pRHO/pPKC | This study |

Table S2. Primers used in this study.

| Name | Sequence (5’-3’) |
| --- | --- |
| cel3a-f | AAATCTATAACTACAAAAAACACATACAG**GAATTC**ATGTTGATGATAGTACAGC |
| cel3a-r | GGGAGATCCTAGCTAGCTAGATCCATGGT**GAATTC**TCACTTGTCATCGTCGTCC |
| Cel5a-f | AAATCTATAACTACAAAAAACACATACAGGAATTCATGAACAAGTCCGTGGCTCC |
| Cel5a-r | GGGAGATCCTAGCTAGCTAGATCCATGGTGAATTCCTACTTTCTTGCGAGACACGAG |
| rho1-f | ATAGCAATCTAATCTAAGTTTTAATTACAAA**GGATCC**ATGTCACAACAAGTTGGTAAC |
| rho1-r | ATCGATTTCAATTCAATTCAATCCTGCAG**GTCGAC**CTATAACAAGACACACTTCTTC |
| pkc1-f | TAGCAATCTAATCTAAGTTTTAATTACAAA**GGATCC**ATGAGTTTTTCACAATTGGAGC |
| pkc1-r | TATCGATTTCAATTCAATTCAATCCTGCAG**GTCGAC**TCATAAATCCAAATCATCTGGC |
| cela-f | AAATCTATAACTACAAAAAACACATACAG**GAATTC**ATGCTTTTGCAAGCTTTCCT |
| cela-r | GGGAGATCCTAGCTAGCTAGATCCATGGT**GAATTC**CTAATAAGGTAGGTGGGGTATG |
| hac1-f | CTTTGTCGCCCAAGAGTATGCG |
| hac1-r | GTGATGAAGAAATCATTCAATTCAAATG |
| Actin-f | CAAACCGCTGCTCAATCTTC |
| Actin-r | AGTTTGGTCAATACCGGCAG |
| Cel3a-F | CCCAGTCTCCATCCCAGAGA |
| Cel3a-R | CAACCGGTACCAGTGGTCAA |
| Sec61-f | TTCCTGCCGGAAGTGATTGC |
| Sec61-r | ACAGAGGGTCGGAAGTCTCA |
| Ssa1-f | CTTCCTCCGCTCAAACTTCCG |
| Ssa1-r | ACCACCGACCAAGACAATTTCATC |
| Kar2-f | CACATTTGCACTTGACGCTAA |
| Kar2-r | AGATTCAACCTTGGCCTTGAT |
| Pdi1-f | GCCATCCACGACATGACTGA |
| Pdi1-r | CTTGGGACTTCACGATTGGG |
| Der1-f | CGGTGCCATTGATGTTTCAC |
| Der1-r | TGTTGCGGAACCAGTCGTAC |
| Hrd3-f | TTCCGTTCCATCTTCTACAG |
| Hrd3-r | CTGGCATCCTCGTCATCTTC |
| Bos1-f | TATGTTTGATCGCCGGAGGC |
| Bos1-r | AGAAATAGGGGCGGTCACAG |
| Erv25-f | TCCACGGCATTCGACGTTTG |
| Erv25-r | TCCACTCTACGCAACTCCAC |
| Snc2-f | GAGTAACTCAGGCGCAAACC |
| Snc2-r | TTTCACCACGTTCAGCAACC |
| Sso1-f | TCAGGCGGAAAACTCCAGAC |
| Sso1-r | GGTGGCCTCTGGTTGAATGA |
| Pir4-f | GTGGTATCCTGACTGACGGT |
| Pir4-r | AGTATCCTGGTCACCGATGG |
| Cwp2-f | ATTCTCTACTGTCGCTTCCG |
| Cwp2-r | GGTGCTGGATGGAGAAACAG |
